# Uterine Fibroids Causing Preterm Birth: A New Pathophysiological Hypothesis on the Role of Fibroid Necrosis and Inflammation

**DOI:** 10.3390/ijms23158064

**Published:** 2022-07-22

**Authors:** Emma E. Don, Anadeijda J. E. M. C. Landman, Guus Vissers, Ekaterina S. Jordanova, Emiel D. Post Uiterweer, Christianne J. M. de Groot, Marjon A. de Boer, Judith A. F. Huirne

**Affiliations:** 1 Department of Obstetrics and Gynaecology, Amsterdam UMC, Vrije Universiteit Amsterdam, De Boelelaan 1117, 1081 HV Amsterdam, The Netherlands; a.landman@amsterdamumc.nl (A.J.E.M.C.L.); guusvissers@gmail.com (G.V.); cj.degroot@amsterdamumc.nl (C.J.M.d.G.); m.deboer2@amsterdamumc.nl (M.A.d.B.); j.huirne@amsterdamumc.nl (J.A.F.H.); 2 Amsterdam Reproduction and Development, Amsterdam, The Netherlands; 3 Center for Gynecologic Oncology, Amsterdam UMC, Vrije Universiteit Amsterdam, De Boelelaan 1117, 1081 HV Amsterdam, The Netherlands; e.jordanova@amsterdamumc.nl; 4 Department of Urology, The Netherlands Cancer Institute/Antoni van Leeuwenhoek Hospital, Plesmanlaan 121, 1066 CX Amsterdam, The Netherlands; 5 Department of Obstetrics and Gynaecology, Amsterdam UMC, University of Amsterdam, Meibergdreef 9, 1105 AZ Amsterdam, The Netherlands; e.d.postuiterweer@amsterdamumc.nl; 6 Department of Obstetrics and Gynaecology, University Medical Center Utrecht, Heidelberglaan 100, 3584 CX Utrecht, The Netherlands

**Keywords:** degeneration, inflammation, necrosis, pregnancy, premature labor, preterm birth, uterine fibroid

## Abstract

According to recent studies and observations in clinical practice, uterine fibroids increase the risk of preterm birth. There are several theories on the pathogenesis of preterm birth in the presence of fibroids. One theory proclaims that fibroid necrosis leads to preterm birth, though pathophysiological mechanisms have not been described. Necrotic tissue secretes specific cytokines and proteins and we suggest these to be comparable to the inflammatory response leading to spontaneous preterm birth. We hypothesize that fibroid necrosis could induce preterm parturition through a similar inflammatory response. This new hypothesis generates novel perspectives for future research and the development of preventative strategies for preterm birth. Moreover, we emphasize the importance of the recognition of fibroids and especially fibroid necrosis by clinicians during pregnancy.

## 1. Introduction

Uterine fibroids are the most common pelvic neoplasms in women of reproductive age, with a prevalence ranging between 5% and 69% [1]. The most common symptoms are excessive menstrual bleeding, menstrual pain, and non-cyclic mechanical complaints [2,3,4]. Symptoms depend on the number, size, and localization of the fibroids, which can be subdivided into submucosal, intramural, transmural or subserosal [1]. A more detailed classification of fibroid location is defined by the International Federation of Gynaecology and Obstetrics (FIGO) classification [5]. Risk factors for fibroids are age, ethnicity, a family history of fibroids, a long interpregnancy interval, and an early menarche. Higher parity and use of hormonal contraceptives are protective factors [1,6].

Women with fibroids more frequently experience reproductive related problems; the chances of natural conception are lower and the miscarriage rate is higher compared to women without fibroids. However, chances and incidence rates vary widely depending on fibroid characteristics, in particular their localization [7,8,9]. During pregnancy, uterine fibroids are present in 2–11% of women and can grow excessively due to increasing estrogenic levels [10,11]. As a consequence of this growth, fibroids can grow faster than neovascularization can follow. This may lead to fibroid degeneration in combination with the formation of thrombosis (so-called red degeneration), leading to necrosis and abdominal pain. Fibroid necrosis is a specific pattern of irreversible, uncontrolled cell death leading to the breakdown of muscle cells and bundles and can even liquefy the fibroid [12,13,14]. When symptoms are severe, a myomectomy can be performed during pregnancy. In this case, Spyropoulou et al. found that most removed fibroids were subserosal fibroids [10]. 

A leading cause of perinatal morbidity and mortality is preterm birth [15]. Globally, preterm birth occurs in 5–18% of all pregnancies, of which two-third occur spontaneously and the remainder are iatrogenic because of maternal or fetal complications [16]. Preterm birth can be sub-categorized depending on gestational age: extremely (<28 weeks), very (28–<32 weeks), and moderate or late (32–<37 weeks) preterm birth [17]. Fetal survival rates depend on gestational age; about 75% of perinatal deaths occur after preterm birth, with two thirds in infants delivered extremely or very preterm (<32 weeks) [18]. Multiple reviews have shown that women with fibroids have an increased risk for preterm birth; however, the underlying pathophysiological mechanisms involved are not clear [7,19,20]. It is important to identify and understand the mechanisms of risk factors, such as fibroid necrosis, in order to improve antenatal care for women who are at risk for preterm birth.

We hypothesize that fibroid necrosis may trigger an inflammatory response leading to spontaneous preterm birth. This pathway could resemble the inflammatory response seen in spontaneous preterm birth. Understanding this necrosis-induced inflammatory pathway is a first and essential step to develop preventative strategies for spontaneous preterm birth in women with fibroid necrosis. First, we illustrate the pathophysiology of inflammation-induced spontaneous preterm birth, after which we demonstrate the similarities to the necrotic pathway. 

## 2. Inflammation-Induced Preterm Birth

During labor, irrespective of gestational age, an inflammatory activation ensues which is self-propagating. A specific balance in pro- and anti-inflammatory factors induces adequate timing of cervical ripening, rupture of the membranes and uterine contractions, which are key events to achieve a normal term delivery [21]. In preterm labor, an early activation of similar processes is initiated by a pathological trigger. However, the nature and scale of the inflammatory cascade may be altered or abnormally high, and the interaction between tissues could be disturbed [21,22,23]. Both human and animal models have shown that spontaneous preterm birth can be triggered by intrauterine inflammation, which can be caused by an intrauterine infection or other infections like appendicitis, pneumonia or gingivitis [15,16]. Differentiation between inflammation caused by an infection or by fibroid necrosis can be challenging during pregnancy. However, after obtaining a medical history and performing physical examination, ultrasonic examination, urine and blood testing, it is possible to exclude inflammatory reactions due to infection. Most important is the attentiveness of the clinician to include fibroid necrosis in the differential diagnosis [24,25].

The oxytocin receptor plays a pivotal role in the initiation of normal parturition, as this receptor is increasingly present in the myometrium of the uterus around the onset of labor [26,27,28]. In late pregnancy, the expression of oxytocin receptors is dramatically increased, and pharmacological inhibitions of this receptor delays delivery [28,29]. The upregulation of oxytocin receptors renders the uterus more susceptible to oxytocin stimulated uterine contractions. After the onset of term or preterm labor, oxytocin receptor levels are maximal and significantly higher compared to before the onset of labor [30]. 

Prostaglandins (PGs), specifically PG-E_2_ and PG-F_2__α_, also have a central role in initiating labor in all phases of parturition [31]. They induce cervical ripening and rupture of membranes by the degradation of collagen and the remodeling of the extracellular matrix [32,33,34]. PGs also directly stimulate contractions of uterine smooth muscle cells [33,35,36]. During inflammation-induced preterm birth, chorionic prostaglandin 15-hydroxy dehydrogenase (PGDH), the primary enzyme deactivating PGs, is decreased in the chorion, allowing PGs to diffuse or transport and facilitate myometrial contractions [37]. Additionally, oxytocin stimulates PG release in the decidua, amnion, chorion and uterine epithelium via cytosolic phospholipase A2 (cPLA2), cyclooxygenase (COX)-1, and COX-2 activation [28,29]. To induce labor by means of intervention, oxytocin and prostaglandins are widely-used therapeutics, both alone and in combination with other interventions [38,39,40].

The high mobility group 1 protein (HMGB1) seems to play a crucial role in inflammatory induced spontaneous preterm birth [29]. Evaluation with Enzyme-linked ImmunoSorbent (ELISA) techniques, polymerase chain reaction (PCR) coupled with electrospray ionization mass spectrometry of sterile amniotic fluid and immunohistochemistry of fetal membranes showed that higher HMGB1 levels are related to preterm delivery [41,42]. By using reverse transcription PCR and ELISA techniques, elevated levels of HMGB1 were again detected in amniotic membranes and fluid of samples after preterm birth and preterm prelabor rupture of membranes (PPROM). Subsequently, the HMGB1 transcripts interleukin(IL)-1β and IL-6 were also increased [43]. Moreover, a direct and significant correlation was found between HMGB1 and IL-6 in amniotic fluid [41]. In mice, the injection of HMGB1 into the amniotic cavity led to preterm birth and higher neonatal mortality within the first week [44]. Elevated levels of IL-6 in maternal plasma, amniotic and cervico-vaginal fluid have been associated with preterm birth in both symptomatic and asymptomatic patients compared to patients who delivered at term [45,46]. Importantly, IL-6 and IL-1β upregulate oxytocin receptors [47,48,49]. 

Next to HMGB1, NF-κB pathway activation is associated with the onset of labor in both term and preterm births [50]. The oxytocin receptor contains transcription factor binding sites for nuclear factor-κB (NF-κB) [47]. During labor, the NF-κB pathway activation leads to upregulation of oxytocin receptors and the transcription of several inflammation associated genes, e.g., IL-1β, IL-8, tumor necrosis factor (TNF)-α, and COX-2, which was found by using a multiplex mass spectrometry method [51]. COX-2 is the principal initiator of PG synthesis in amniotic tissue. Together with TNF-α, IL-1β leads to an increase in COX-2 expression and consequently to increased PG production [51].

Balance in reactive oxygen species (ROS) and antioxidants is important for tissue homeostasis, and an imbalance can lead to oxidative stress. In the uterus, excessive production of ROS may result in pregnancy complications such as preeclampsia, fetal growth restriction and preterm birth. ROS are an inseparable component of inflammation, as they can regulate inflammatory cytokines [52,53]. The review by Moore et al. describes that in most studies measuring oxidative stress, ROS or their byproducts were increased and antioxidants were decreased in preterm specimens compared to specimens after term birth [53].

Recently, microRNAs (miRNAs) have been found to be involved in parturition by influencing hormone responsiveness, regulating key gene expression pathways, and increasing myometrial sensitivity to oxytocin [28,54]. For instance, the miRNA-family 200 modulates expression of the oxytocin receptor and connexin-43 (CX-43) via Zinc finger E-box-binding homeobox (ZEB)1 and ZEB2 [55]. ZEB1 and ZEB2 can inhibit the expression of the oxytocin receptor and CX-43, thus inhibiting myometrial contractions [55]. A mouse model demonstrated that, during preterm birth, miRNA200 expression was upregulated and ZEB1 and ZEB2 expression was downregulated [55]. However, when comparing miRNAs between women who delivered at term or preterm, no significant differences were found in serum values [56]. Yet, after profiling miRNAs in cervical cells, 99 miRNAs were different between samples of patients after preterm delivery compared to term delivery, and the expression of miRNA-143 and -145 differed the most [57].

The process of inflammation-induced preterm birth is complex, and more pathways are involved which we have not all described in this narrative review. Nevertheless, we have attempted to describe the most important pathways which could possibly be comparable to the inflammatory response leading to spontaneous preterm birth after fibroid necrosis.

## 3. Fibroid Necrosis Initiated Spontaneous Preterm Birth

The timing of the formation of the first fibroid is not known. However, clinical data indicate that fibroids are rare during the early reproductive years. Because the majority of women have asymptomatic fibroids, these can typically be diagnosed during the first routine ultrasound during pregnancy [25]. During pregnancy, especially in the first trimester and early second trimester, some fibroids grow extensively [10,58,59,60]. Because of this extensive growth a fibroid can overgrow its own blood supply, leading to inadequate oxygenation and, consequently, necrosis [13]. The incidence of necrosis during pregnancy varies from 2% to 28% in patients with fibroids [24]. The incidence of hospitalization because of fibroid related pain during pregnancy is 5–15%. This risk increases with the size of the fibroid and is high if the fibroid diameter is >5 cm. However, it is unknown whether fibroid related pain corresponds directly with fibroid necrosis [12,61]. Hypothetically, the faster the growth of the fibroid (thus the larger the fibroid) the larger the chance of outgrowing its own blood supply and subsequent necrosis. Additionally, pedunculated subserosal fibroids (FIGO type 7) can possibly twist their own stalk, cutting of blood supply acutely, resulting in necrosis. Although not specifically studied in (pregnant) women with fibroids, necrosis is generally known to trigger inflammation through various cellular and molecular reactions. Consequently, when fibroids necrotize, they activate an inflammatory cascade. We will focus on the parallelism of reactions after general cell necrosis and the inflammatory pathway studied in patients after spontaneous preterm birth. 

Immunoblotting and immunostaining demonstrated that HMGB1 is passively released during necrosis, promoting inflammation by stimulating the production of TNF-α. Conversely, apoptotic cells do not excrete HMGB1, even after secondary necrosis or partial autolysis, and therefore are unable to inducing inflammation via this pathway [62]. Furthermore, HMGB1 is essential for sterile inflammation following injury and activates the recruitment of neutrophils, leading to necrosis [63].

Parallel to the release of HMGB1, necrotic cells activate the NF-κB pathway by the secretion of heat shock proteins (HSPs) [64]. HSPs are produced after cell injury, and important HSP subtypes are HSP70, HSP90, and GP96 [64,65]. HSP production is induced by heat shock factor 1 (HSF1) and hypoxia-inducible factor 1α (HIF-1α), where HIF-1α regulates HSF1, and HSF1 regulates HSP release [66,67]. When HSPs are released in the extracellular milieu, they activate the NF-κB pathway, which is also not observed after apoptotic cell death but only in necrosis [64]. Activation of the NF--κB pathway subsequently leads to the upregulation of transcription of several inflammation-associated genes, e.g., IL-1β, IL-8, TNF-α, and COX-2 [47,50,68]. Since we hypothesize that after fibroid necrosis the TNF-α pathway could be triggered, the former may rather be considered necroptosis, as necroptosis is a programmed form of necrosis and is typically seen as a response to TNF pathway activation [69].

Necrosis can be the result of oxidative stress, and the source of this is not ROS per se but the imbalance of ROS production and detoxification [70]. ROS can be produced in the mitochondria, and studies show that there is a complex cross-play between ROS and TNF-α in the inner mitochondrial membrane, influencing necrotic cytotoxicity [70,71]. Moreover, intracellular ROS is involved in NF-κB signaling and the downstream production of TNF [71].

MiRNA-145 was found to be upregulated in steroid-induced necrosis of the femoral head [72]. Furthermore, miRNA-145 accelerates the NF-κB pathway activation as well as the expression of IL-1β, TNF-α in atherosclerosis cells in mice [73]. Additionally, in liver fibrosis samples, miRNA-145 was downregulated and ZEB2 expression was upregulated [74]. No specific studies were found investigating miRNA-143 or -200 in necrotic cells. However, in one study focusing on the pathogenesis of H. pylori-associated gastritis, a positive correlation was found between the miRNA-200 family and IL-1β, IL-6, or TNF-α expression in gastric mucosa [75].

**Figure 1:**
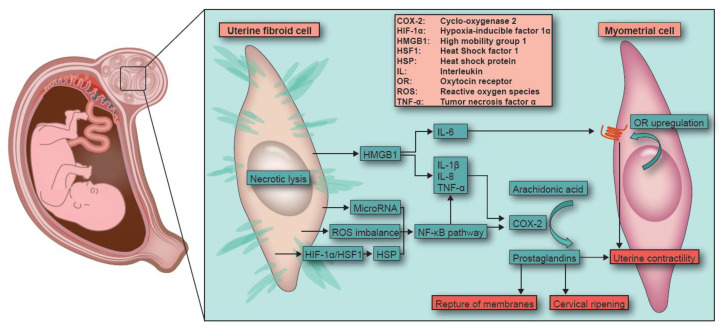
Fibroid necrosis during pregnancy. Schematic representation of the hypothesized inflammatory pathway of necrosis and induction of pre-term labor in women with fibroid necrosis during pregnancy.

These pathways, all activated during necrosis, could hypothetically also be activated during fibroid necrosis in pregnancy, where they can lead to the upregulation of oxytocin receptors and cause an increase in PG production in myometrial cells (schematically depicted in Figure 1). This could induce spontaneous preterm birth by stimulating uterine contractility, cervical ripening and rupture of the membranes. 

It is likely that other inflammatory pathways could also play a role in necrosis-induced spontaneous preterm birth, and our hypothesis is a simplification of an extensive and complex interaction of factors. Nevertheless, it highlights the link between some of the most studied parameters associated with preterm birth.

## 4. Future Perspectives

To test our hypothesis, we propose a study in which women with (necrotic) fibroids are prospectively followed during pregnancy and compared to women without fibroids. Blood serum and plasma of the patient could be tested by multi-parameter techniques, like a Luminex or MesoScale, focusing on necrosis associated interleukins and TNF-α [76]. Samples could be collected at the moment of first presentation with fibroid related pain, during and after (preterm) birth, and placentas, amniotic fluid and fetal membranes could also be collected to study inflammatory parameters. In addition, oxytocin receptor expression levels can be compared between necrotic and vital myometrial cells using reverse transcriptase PCR. Furthermore, HMGB1, HSP and specific miRNAs expression levels could also be compared between necrotic and vital tissue samples.

Interventions to prevent preterm birth are widely studied [77]. Progesterone and low-dose aspirin (ranging from 75–160 mg daily) are used as preventive pharmacological strategies for preterm birth. Both have anti-inflammatory effects, making it worth investigating whether these interventions could prevent inflammation-induced preterm birth, specifically in women with fibroids or fibroid necrosis. Treatment with progesterone may help by interfering with the NF-κB pathway and reducing the production of TNF-α among other pro-inflammatory cytokines [78]. Low-dose aspirin is a dual COX-inhibitor which may prevent inflammation-induced preterm birth by interfering in the prostaglandin synthesis [79,80,81]. Currently, low-dose aspirin is being evaluated for the prevention of preterm birth in women with adenomyosis (NCT04535804) based on an increased inflammatory environment of adenomyosis that is closely related to the initiation of preterm birth [82].

Anti-TNF-α does not seem to affect preterm birth risk, although this has only been studied in an observational setting in patients who used anti-TNF-α because of inflammatory bowel disease [83]. It would be interesting to study whether anti-TNF-α could prevent preterm birth in patients with fibroids. Furthermore, Moylan et al. showed that myometrial activity was prevented by short chain fatty acids suppressing the NF-κB pathway in an in vitro model of preterm birth [84]. Gomez et al. showed that the inhibition of the HSP NLRP3 via MCC950 prevented preterm birth and reduced perinatal mortality in a mouse model [85]. Several studies found therapeutics to bind HMGB1 to inhibit its effect [86,87,88]. Although these study results are too premature to implement in daily clinical care, they motivate future research to ultimately bring treatments from bench to bedside. 

Ideally, we would be able to identify patients at risk for developing fibroid necrosis during early pregnancy or even antepartum. With the development of a prognostic model based on uterine fibroid characteristics, we might be able to predict which patient is at risk for fibroid necrosis during pregnancy and consider myomectomy before conception. However, no preventive therapy exists for fibroids. Nonetheless, research on dietary interventions such as prophylaxis, like vitamin D supplementation, seems promising but still needs to be tested in randomized controlled trials [89].

In conclusion, we hypothesize that fibroid necrosis can lead to spontaneous preterm birth through a similar pathway as inflammation-induced spontaneous preterm birth. A better understanding of the pathophysiological mechanisms causing preterm birth in pregnancies complicated by fibroid necrosis is essential to develop strategies to prevent this unfavorable outcome in women with uterine fibroids.
